# Mitochondria in Cardiac Postconditioning

**DOI:** 10.3389/fphys.2018.00287

**Published:** 2018-03-26

**Authors:** Pasquale Pagliaro, Saveria Femminò, Jasmin Popara, Claudia Penna

**Affiliations:** Department of Clinical and Biological Sciences, University of Turin, Turin, Italy

**Keywords:** cardioprotection, ischemia/reperfusion, reactive oxygen species, redox signaling, mitochondria, connexin 43

## Abstract

Mitochondria play a pivotal role in cardioprotection. Here we report some fundamental studies which considered the role of mitochondrial components (connexin 43, mitochondrial KATP channels and mitochondrial permeability transition pore) in postconditioning cardioprotection. We briefly discuss the role of mitochondria, reactive oxygen species and gaseous molecules in postconditioning. Also the effects of anesthetics—used as cardioprotective substances—is briefly considered in the context of postconditioning. The role of mitochondrial postconditioning signaling in determining the limitation of cell death is underpinned. Issues in clinical translation are briefly considered. The aim of the present mini-review is to discuss in a historical perspective the role of main mitochondria mechanisms in cardiac postconditioning.

## Introduction

Mitochondria are fundamental as sources of energy, but also to sustain life being elements involved in cell survival and death. Mitochondrial dysfunction is a critical element of many diseases including ischemia/reperfusion (I/R) and subsequent development of ventricular systolic dysfunction and possible compensatory heart hypertrophy. This article outlines the role of mitochondria as targets for reducing I/R damage in myocardial postconditioning.

Cardiac postconditioning has been defined by the seminal work of Vinten-Johansen's group as “repetitive ischemia applied during early reperfusion” (Zhao et al., 2003). The name postconditioning was proposed in comparison with the previously discovered ischemic preconditioning. It soon became clear that intramyocardial mechanisms are responsible for both pre and postconditioning cardioprotection and that mitochondria may play a pivotal role (Pagliaro et al., 2004; Tsang et al., 2004; Hausenloy and Yellon, 2016).

Postconditioning attracted the interest of researchers as it allows an easier approach in humans. Indeed, it has been tested several times, both with a mechanical (brief ischemia) or pharmacological approach to target mitochondria in animals and humans. Also, inhibition of mitochondrial permeability transition to limit the so-called “post-cardiac arrest syndrome,” observed in patients resuscitated from cardiac arrest, has been tested in a pre-clinical study (Cour et al., 2011). The results with both approaches are contradictory and have been reviewed elsewhere (Gomez et al., 2009; Penna et al., 2013a; Dongworth et al., 2014). The main purpose of the present article is a diachronic approach to studies that considered mitochondria mechanisms involvement in postconditioning.

## Mitochondria and heart postconditioning

Searching on Pubmed for “Mitochondria^*^[title] and heart and postconditioning” with a publication date limit from 2003/01/01 to 2017/12/31 we found 82 articles.

In this series of article, the first report which hypothesized and confirmed an important role for mitochondria in postconditioning was the article by Argaud et al. (2005). These authors confirmed a role for the *mitochondrial permeability transition pore* (mPTP) in lethal reperfusion injury and suggested that this pore is modulated by postconditioning. The study was conducted in anesthetized open-chest rabbits. Mitochondria were isolated from the risk area of myocardium, and calcium-induced mPTP opening was determined using a potentiometric method. Postconditioning inhibited the opening of the mPTP and provided a robust anti-ischemic protection. Later, the same group demonstrated that mitochondrial calcium decreased in pre-conditioning, but increased significantly either in postconditioning or after inhibition of mPTP (Argaud et al., 2008). These data have suggested that Ca^2+^ retention within mitochondria may clarify the limitation of reperfusion damage in postconditioned hearts (but not in preconditioned). The involvement of mitochondria in postconditioning protection has been confirmed in several studies, for Reviews see ((Boengler et al., 2011a, 2013; Di Lisa et al., 2011)).

**Figure 1:**
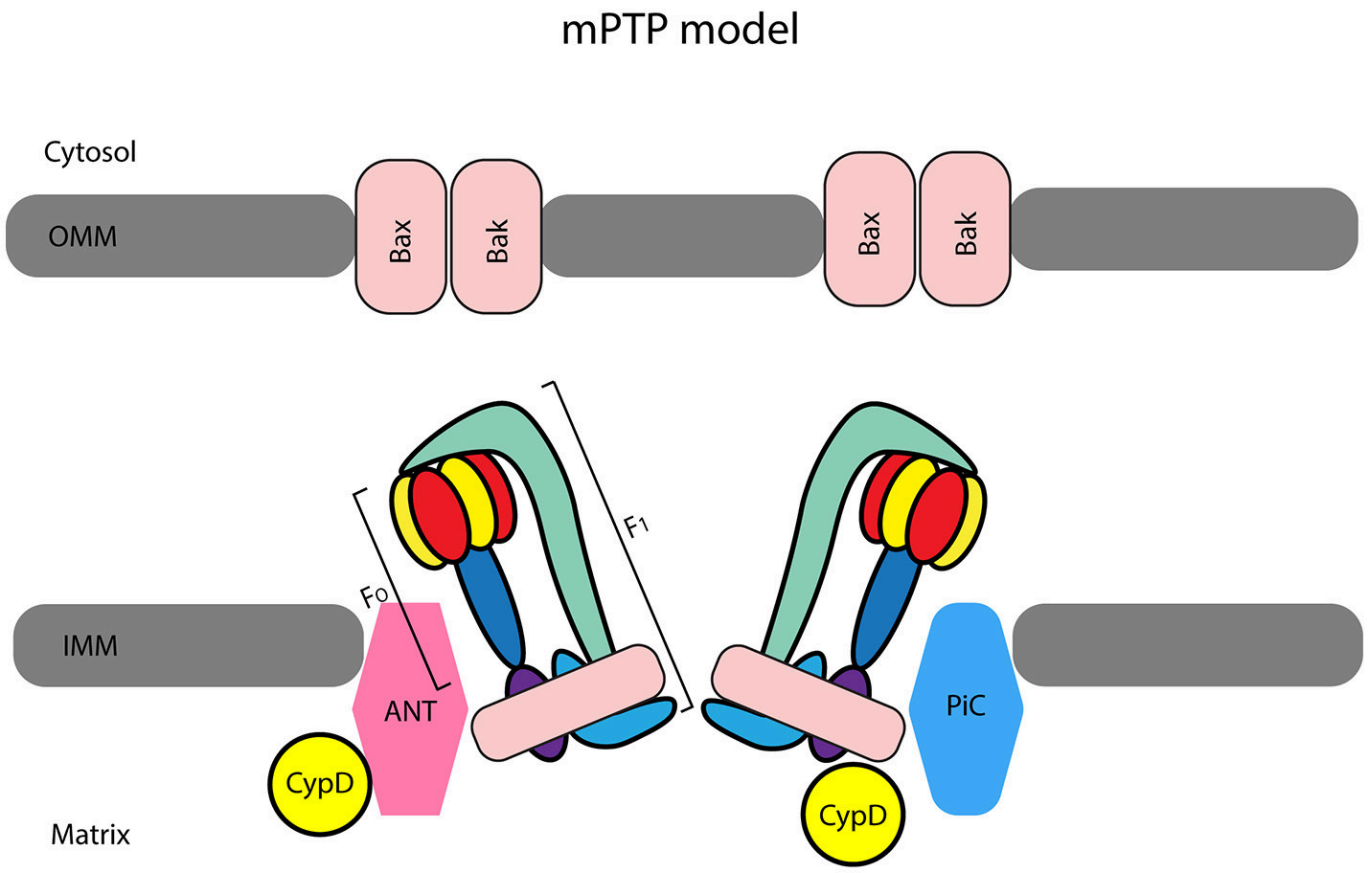
The most accepted model considers ATP synthase subunits as central elements of the mitochondrial permeability transition pore (mPTP). Indeed, the F1FO ATP synthase has been suggested by Bernardi et al. (2015) to be a pore component for the inner mitochondrial membrane (IMM) forming a unit of the mPTP. It has been suggested that adenine nucleotide translocase (ANT) and cyclophilin-D (CypD), together with mitochondrial phosphate carrier (PiC) form a complex that acts as pore regulator. The pro-apoptotic proteins Bax/Bak located in the outer mitochondrial membrane (OMM) favor swelling and subsequently, mitochondria rupture once the IMM complex opens (Kwong and Molkentin, 2015).

The mPTP, whose nature is still controversial (Figure 1), plays a pivotal role in the shift from life to death (Bernardi et al., 2015; Kwong and Molkentin, 2015). Already in 2006 Ovize's group reviewed the evidence for an important role of the mPTP in postconditioning (Gateau-Roesch et al., 2006). It was soon evident that mPTP priming occurs during ischemia and early reperfusion, and that mPTP opens at the time of full reperfusion, leading to cell death, whereas pre- and postconditioning prevent the pore formation. Also, modulation of electron transport has emerged as a mechanism responsible for cardiac mitochondria protection, which decreases myocardial injury during ischemia and early reperfusion (Chen et al., 2006). In 2007 Gomez et al. confirmed that inhibition of mPTP at reperfusion not only limits infarct size but also improves functional recovery and mice survival (Gomez et al., 2007). Then, in a dog model of myocardial I/R, Mykytenko et al. demonstrated that the beneficial effects of postconditioning and effects on mitochondrial function persisted 24 h after the ischemic event (Mykytenko et al., 2008). In particular, postconditioning reduced infarct size and decreased CK activity after prolonged reperfusion and the protection was attributable to the opening of mitochondrial KATP channels (mKATP) and inhibition of mPTP opening. Nevertheless, mPTP physiology is complex and its transient opening during preconditioning is protective (Dongworth et al., 2014; Hausenloy and Yellon, 2016).

It is likely that the *signal transducer and activator of transcription 3* (STAT3) contributes to cardioprotection by stimulation of respiration and inhibition of mPTP opening (Boengler et al., 2010; Heusch et al., 2011). We confirmed the role of STAT3 in ischemic postconditioning but as a component upstream to mitochondrial ROS (Reactive Oxygen Species) signaling (Penna et al., 2013b). In cardiac cells, two main types of mitochondria are present: interfibrillar (IFM) and subsarcolemmal (SSM) with different biochemical and morphological properties (e.g., lower oxidation potential and lower enzyme activities of complex I, succinate dehydrogenase, in SSM than IFM) (Palmer et al., 1977). In cardiac cells, STAT3 was principally present in the matrix of SSM and IFM. STAT1 was also found in mitochondria under physiological conditions, but this does not occur for STAT5 (Boengler et al., 2010; Heusch et al., 2011).

Mitochondrial *connexin 43* and postconditioning protection were studied several times (Penna et al., 2009; He et al., 2010; Boengler et al., 2011a; Di Lisa et al., 2011). The mitochondrial location of connexin 43 being central for cardioprotection has been proposed by Schulz and co-workers (Boengler et al., 2011a). It is clear that mitochondrial connexin 43 has a role in postconditioning-induced ROS-signaling, but its precise function is not clear. Recently, Tu et al. (2017) have described a role for mitochondrial connexin 43 in hypoxic postconditioning. However, postconditioning (unlike preconditioning) effectively reduces infarct size in heterozygous connexin 43-deficient (Cx43^+/−^) mice *in vivo* (Heusch et al., 2006), questioning the role of connexin 43 in this cardioprotective intervention. Nevertheless, a difference exists between IFM and SSM in terms of connexin 43 presence and function. The role of these subpopulations of mitochondria deserves more studies.

The *mitochondrial ATP-sensitive K*^+^ *channels* (mKATP) have a putative important role in postconditioning cardioprotection (Garlid and Halestrap, 2012; Jin et al., 2012). It has also been suggested that the mitochondrial calcium uniporter is involved in the mechanisms of ischemic postconditioning (Yu et al., 2011).

## Mitochondria and ROS

Serviddio et al. suggested that mitochondria play a pivotal role in H_2_O_2_ production and redox stress during reperfusion and are important for the cardioprotective effect of postconditioning (Serviddio et al., 2005). These authors used isolated perfused rat hearts in which they compared an early normoxic reperfusion with a hypoxic reperfusion. They found out that mitochondria carbonyl proteins are somewhat lower in hypoxic than in the normoxic group at the end of reperfusion and concluded that hypoxic reperfusion at its onset limits myocardial injury and the amount of mitochondrial H_2_O_2_ production. Although this was not a real postconditioning, as defined by Vinten-Johansen's group (Zhao et al., 2003), it was the first study to hypothesize some changes in redox aspects within mitochondria in early reperfusion after a protective intervention.

We were the first to show that in order to induce cardioprotection by postconditioning in isolated perfused rat hearts, a signaling through a mKATP activation and redox-sensitive mechanism is required (Penna et al., 2006). It is likely that postconditioning procedures reduce the production of ROS in early reperfusion, but if ROS are completely removed in the initial minutes of reperfusion the heart cannot be protected by the “repetitive ischemia applied during early reperfusion.” Our observation was confirmed several times by different laboratories. The same year Bopassa et al. confirmed the involvement of mPTP and suggested that *phosphatidylinositol 3-kinase* (PI3K) regulates mPTP in isolated perfused rat hearts subjected to a postconditioning protocol (Bopassa et al., 2006). We then demonstrated that targeting of specific cellular sites such as *bradykinin B2 receptors* and mKATP channels during early reperfusion elicits postconditioning-like protection through ROS signaling and ROS compartmentalization (Penna et al., 2007). Very recently Boengler et al. (2017) have shown that p66shc is present in both SSM and in IFM. However, it seems that ROS formation by p66shc is not involved in determining myocardial injury.

## Mitochondria and gaseous cardioprotective substances

Hydrogen sulfide (H_2_S), carbon monoxide (CO) and Nitric Oxide (NO) are recognized as three gaseous mediators for cardioprotection. All these molecules have potential cardioprotective effects in the heart. In particular, the beneficial actions were demonstrated against myocardial I/R injury, including infarction, arrhythmia, hypertrophy, fibrosis, and heart failure. These protective effects were mediated by complex pathway and the effects included: anti-oxidative action, anti-inflammatory responses, reduction of apoptosis, angiogenic actions, and regulation of ion channel (Mancardi et al., 2009; Andreadou et al., 2015; Penna et al., 2015). Since these gasses may be produced within mitochondria and may profoundly affect the function of these organelles, here we discuss briefly their role in the context of cardioprotection. The enzymatic production of NO is mediated by three isoforms of NOS isoforms: neuronal (nNOS or NOS I), inducible (iNOS or NOS II), and endothelial (eNOS or NOS III). A specific mitochondrial NOS isoform has been also proposed. NOS activity is governed by different factors (co-factors and substrate availability, and endogenous inhibitors) and the NO can induce post-transcriptional, post-translational and transcriptional modulations in specific subcellular compartments. Importantly, during ischemia, when pH becomes acidic and oxygen-dependent NOS activity may be impaired, the formation of NO can also derive from the non-enzymatic reduction of nitrite/nitrate, which can be dietary and endogenous in origin (Andreadou et al., 2015).

Also, the activation of the NO/cGMP pathway, with augmentation of cGMP and NO levels, has been observed after postconditioning maneuvers in different cardiac models. During postconditioning, the activation of eNOS and Akt pathway converge on Glycogen Synthase Kinase-3β (GSK-3β) and inhibits mPTP opening (Correa et al., 2015). Different concentrations of NO induce different action in the mitochondria. In particular, high NO concentration open the mPTP with the formation of peroxynitrite and disulphide bonds formation, while physiological NO levels favor mPTP closure with post-translational modification of protein S-nitrosylation (Correa et al., 2015 and references therein). In early reperfusion, a temporary interruption of respiration may prevent exaggerated generation of superoxide anion (${\text{O}}_{2}^{-}$) and ONOO^−^ and reduce the thiol oxidation with permanent inactivation of metabolic enzymes or inhibition of mPTP opening (Piantadosi, 2012).

Hydrogen sulfide (H_2_S) is produced by several enzymes, within and outside mitochondria. It seems that H_2_S produced by cystathionine-gamma-lyase (CSE) from L-cysteine can readily scavenge the ROS and may induced protection with two mechanisms, one reperfusion injury salvage kinase (RISK)-dependent and the other RISK-independent. Therefore, H_2_S as NO has important antioxidant properties, but in contrast to NO, H_2_S cannot directly form radicals (Mancardi et al., 2009). An interesting and recent paper by Banu et al. (2016) reports that both postconditioning maneuvers and H_2_S postconditioning significantly restores the complex I activity to near normal level, particularly in IFM. The preserved IFM activity was evidenced by the improvement in electron transport chain enzyme activities and mitochondrial respiration.

Endogenous carbon monoxide (CO) is synthesized by hemoxygenases (HO-1 and HO-2) as a consequence of the catabolism of haem and is an important bioactive molecule. It has been observed that CO induces the mitochondrial production of ${\text{O}}_{2}^{-}$, which is transformed by superoxide dismutase to H_2_O_2_, and then a subsequent Akt activation by H_2_O_2_ limits apoptosis after I/R (Kondo-Nakamura et al., 2010). Moreover, the anti-apoptotic effects of CO are related to the inhibition of mPTP. In isolated mitochondria, CO inhibited mPTP opening, loss of potential, cytochrome c release and swelling (Queiroga et al., 2010). Further details on the role of NO, H_2_S, and CO in cardioprotection can be found on Andreadou et al. (2015).

## Mitochondria and anesthetics

Agents targeting mitochondria with prominent postconditioning effects are anesthetics. The volatile anesthetic *sevoflurane* given for 2 min at the beginning of reperfusion-induced myocardial protection against myocardial I/R injury. This sevoflurane-postconditioning is mediated, at least in part, by mKATP-channels (Obal et al., 2005). Almost simultaneously, in a similar model, Feng et al. have published that another volatile anesthetic, *isoflurane*, induces postconditioning preventing the opening of the mPTP *via* inhibition of GSK-3β (Feng et al., 2005). It was also demonstrated, *in vivo*, that the antiapoptotic protein B cell lymphoma-2 (Bcl-2) mediates myocardial postconditioning protection by isoflurane, thus indirectly modulating mPTP activity (Wang et al., 2006; Pravdic et al., 2010).

*Propofol*, another anesthetic, also displayed cardioprotective effect against cardiac I/R injury associated with inhibition of mPTP opening. Intriguingly, compared to propofol, sevoflurane induces more beneficial effects on functional recovery and infarct size (He et al., 2008). Another study suggested that sevoflurane postconditioning protects isolated rat hearts through the involvement of the ROS-ERK 1/2-mPTP signaling cascade (Yao et al., 2010). Moreover, sevoflurane postconditioning protects infarcted rat hearts against I/R damage by inhibiting mPTP opening through the involvement of PKB/Akt and ERK1/2 (Yao et al., 2009). Nevertheless, sevoflurane-induced postconditioning, as other conditioning protocols, results impaired by the presence of hyperglycemia. This impairment of protection was reversed by the mPTP inhibition with cyclosporine A (Huhn et al., 2008) or by inhibition of excess mitochondrial fission with dynamin-related protein 1 inhibitor (Yu et al., 2017). Lim et al. have confirmed that the mPTP plays an essential role in in the cardioprotection induced by ischemic and pharmacological preconditioning and by postconditioning (Lim et al., 2007). Yet, pharmacological postconditioning may be limited by a “ceiling effect of protection,” but, this ceiling effect may be reversed by simultaneous inhibition of GSK-3β *via* the opening of mKATP channels (Couvreur et al., 2009). GSK-3β modulates mitochondrial function and Gomez et al.confirmed that GSK-3β inhibition *via* its S9-phosphorylation is required for postconditioning and that this phosphorylation likely works by inhibiting the opening of the mPTP (Gomez et al., 2008). Indeed, it has been suggested that the phosphorylation/inactivation of GSK-3β is involved in the inhibition of mPTP opening *via* the interaction with several elements of the mPTP regulatory complex and subsequent increase in mPTP-ROS threshold (Tanno et al., 2014). Finally, it is of note that *morphine*, an opiate often given to patients who have undergone surgery and anesthesia, may induce postconditioning *via* delta-1 opioid receptors activation and mPTP modulation (Kim et al., 2011).

## Mitochondrial postconditioning signaling and limitation of cell death

Postconditioning signaling converges on mitochondria, thus limiting all forms of cell death. We have suggested that postconditioning or perfusion of the heart with bradykinin may activate cellular signaling leading to the opening of mKATP channels, increasing ROS production, inhibiting the mPTP and inducing cardioprotection (Penna et al., 2006). It has been suggested that the cooperation between bradykinin and bradykinin-receptor may favor the assembly of a caveolar signaling platform (*signalosome*). The receptors with ligands migrate to caveolae, where signaling elements are scaffolded into signalosomes that translocate to mitochondria. The signalosome-mitochondria interaction then initiates mKATP channels, increases ROS production, which favors mitochondrial protein kinase C epsilon activation and mPTP inhibition, thus decreasing myocardial injury (Quinlan et al., 2008). It has been suggested that postconditioning similarly to adenosine may induce HSP90-dependent translocation of PKCε to mitochondria, likely *via* mitochondrial import machinery TOM70 (Yang et al., 2012). These results suggest an important implication of cytosolic protein translocation within mitochondria in ischemic postconditioning (Boengler et al., 2011b).

Mitochondria are important players in many types of *apoptotic* and necrotic cell death (Murphy and Steenbergen, 2011). We were among the first to demonstrate that postconditioning increases the levels of anti-apoptotic markers, including the phospho-GSK-3β and Pim-1 kinases, while decreasing the pro-apoptotic markers, namely cytochrome c, thus preserving the mitochondrial morphology (Penna et al., 2009). Fang et al. confirmed that postconditioning attenuates cardiomyocyte injury and apoptosis by blocking mPTP (Fang et al., 2008). Subsequently, Li et al. suggested that the cardioprotective effect of postconditioning is mediated by apoptosis repressor with caspase recruitment domain (ARC) (Li et al., 2009). Dong et al. also showed that postconditioning may protect cardiomyocytes from apoptosis *via* an interaction between PKCε and calcium-sensing receptors to inhibit endoplasmic and sarcoplasmic reticulum-mitochondria crosstalk (Dong et al., 2010).

The influence of mitochondrial dynamics in I/R and cardioprotection, and their potential as targets in treating cardiovascular disease, are also emerging (Boengler et al., 2011a; Ong and Hausenloy, 2017). Finally, experimental studies highlighted the importance of exosomes and vesicles in local and distant intercellular communication mechanisms after myocardial infarction. Exosomes and vesicles are potentially useful as cell-free therapeutic candidates (Lai et al., 2010; Bell et al., 2012; Chen et al., 2013; Barile et al., 2014; Giricz et al., 2014; Ibrahim et al., 2014; Yellon and Davidson, 2014; de Couto et al., 2017; Sluijter et al., 2018). However, caution must be used and extensive studies are necessary because their mechanisms of protection are still unknown.

## Translation issues

Pharmacological and mechanical ischemic postconditioning can be therapeutic options (Pagliaro and Penna, 2015). For instance, blocking the mPTP could be beneficial, but mPTP blockers have yielded mostly neutral effects in both myocardial infarction and heart failure patients. Also, mechanical ischemic postconditioning yielded contradictory results. In animal models, postconditioning resulted in an increase in myocardial salvage (about 30 % in rats, 35% in dogs, 50% in pigs, and 65% in rabbits) (Zhao et al., 2003; Mykytenko et al., 2008; Sun et al., 2010). However, in humans studies of postconditioning effects on markers of myocardial injury have obtained conflicting results (Lønborg, 2015; Pagliaro and Penna, 2015). Several authors (Staat et al., 2005; Thibault et al., 2007; Xue et al., 2010) reported a decrease in enzyme leakage. Lønborg et al. (2010) using magnetic resonance imaging found an increase in myocardial salvage ratio. Yet authors (Sörensson et al., 2010; Freixa et al., 2012; Hahn et al., 2013) do not observe any effect of postconditioning in humans with myocardial infarction. Thus, additional studies with adequately sized and designed randomized trials are necessary. Hope comes from a recent trial which reports a significant increase in myocardial salvage when classical postconditioning has been combined with remote ischemic conditioning (Eitel et al., 2015).

## Conclusions

*In conclusion*, here we have reported several studies which have shown that different signal transduction pathways are switched on or switched off both by ischemic postconditioning and by pharmacological postconditioning. These signaling pathways converge on mitochondria where different components are affected preserving many of the mitochondrial functions after ischemia/reperfusion. Within mitochondria, a central role is played by connexin 43, mKATP channels and mPTP. Mitochondrial dynamics are also of fundamental importance in I/R and cardioprotection (Boengler et al., 2011a; Ong and Hausenloy, 2017). Many other factors and consequently several other studies are not considered and we apologize to authors of those studies. However, the core aim of the present mini-review was to report the main steps which allow us to understand the role of these organelles in postconditioning and it may represent a starting point to deepen the understanding of mitochondria role in cardioprotection. Future researches and developments in this field should rely on appropriate animal models (with comorbidities and co-medication) that can allow identifying candidates for future clinical trials and, 1 day, discovery the appropriate strategies to eradicate myocardial infarction and its sequela.

## Author contributions

CP and PP drafted the first version and supervised the manuscript. All authors evaluated retrieved papers and their reference lists to identify additional relevant articles. JP and SF made the figure. All authors revised the manuscript and approved the final version of the manuscript.

### Conflict of interest statement

The authors declare that the research was conducted in the absence of any commercial or financial relationships that could be construed as a potential conflict of interest.
